# Effect of age on long-term outcomes after stroke with atrial fibrillation: a hospital-based follow-up study in China

**DOI:** 10.18632/oncotarget.15729

**Published:** 2017-02-25

**Authors:** Tao Wang, Bin Li, Hongfei Gu, Yongzhong Lou, Xianjia Ning, Jinghua Wang, Zhongping An

**Affiliations:** ^1^ Department of Neurology, Tianjin Haibin People’s Hospital, Tianjin, China; ^2^ Department of Neurology, Tianjin Medical University General Hospital, Tianjin, China; ^3^ Department of Epidemiology, Tianjin Neurological Institute, Tianjin, China; ^4^ Tianjin Neurological Institute, Key Laboratory of Post-Neuroinjury Neuro-Repair and Regeneration in Central Nervous System, Ministry of Education and Tianjin City, Tianjin, China; ^5^ Department of Neurology, Tianjin Huanhu Hospital, Tianjin, China

**Keywords:** ischemic stroke, atrial fibrillation, outcomes, risk factors, age

## Abstract

Atrial fibrillation (AF) is an established predictor of poor outcomes after stroke. We aimed to assess the effect of age on outcomes at 1 year and 3 years in stroke patients with AF. We recruited acute ischemic stroke patients with AF between January 2006 and September 2014 in Tianjin, China. Clinical features and outcomes at 1 year and 3 years after stroke were compared between younger group and elderly group. Overall, 951 consecutive stroke patients with AF were included in this study. There was a higher mortality and dependency rate in the elderly group than in the young group at both 1 and 3 years after stroke. Recurrence rates were significantly higher in the elderly group than in the young group at 3 years after stroke. The higher risks of mortality and dependency in elderly patients remained unchanged, but disappeared in recurrence rates after adjusting for stroke subtype, severity, risk factors, and lifestyle. These findings suggest that it is crucial to highlight the treatment of elderly stroke patients with AF in order to reduce poor outcomes and to reduce the burden of AF in China.

## INTRODUCTION

Stroke is the leading cause of death and disability in both developed and developing countries worldwide [1–3]. The incidence of stroke has declined or plateaued in developed countries [4–6], but it has dramatically increased in developing countries, especially in China [7–9]. Although there had been a trend toward the occurrence of incident stroke later in life in the past two decades, the proportion of stroke burden is greater overall in individuals <75 years than in those who are older, especially in low-income and middle-income regions [10].

Atrial fibrillation (AF) is the most common sustained cardiac arrhythmia, which is an established risk factor for ischemic stroke [11, 12]. The prevalence of AF in the general population increases substantially with age, ranging from 0.1% in those aged <55 years to 9% in those aged ≥80 years [13]. Although there is a low prevalence of AF in China, the disease burden of AF-related stroke is great due to the aging population.

Age is the most important non-modifiable risk factor for stroke [14, 15], and elderly patients experiencing stroke generally have poor functional outcomes afterwards [16–18]. Moreover, patients with AF-associated stroke have higher mortality than do stroke patients without AF, with rates between 30.5% and 63.0% at 12 months after stroke [19, 20]. However, the long-term outcomes and risk factors for these outcomes in patients with AF-associated stroke in China are uncertain. We therefore aimed to assess the effect of age on outcomes and the risk factors for these outcomes among patients with AF-associated stroke in China.

## RESULTS

**Figure 1:**
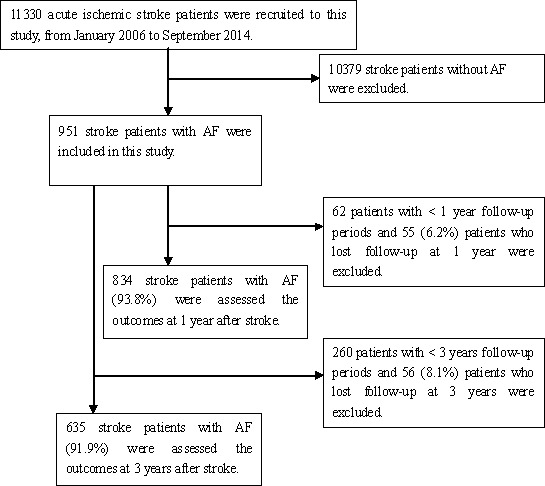
Flow chart of Participants

A total of 11,330 consecutive patients with ischemic stroke treated at three large hospitals in Tianjin during the study period were registered for this study. Of these, 951 patients had AF. Outcomes were assessed for 834 patients with AF-associated stroke at the 1-year follow-up, after excluding 55 patients lost to follow-up and 62 patients with a follow-up period of <1 year; the response rate for follow-up was thus 93.8%. Outcomes for 635 patients were assessed at the 3-year follow-up, after excluding 56 patients lost to follow-up and 260 patients with a follow-up period of <3 years; the response rate for this follow-up was thus 91.9% (Figure 1).

### Differences in clinical features between groups

Overall, 951 patients with AF-associated stroke were included in this study; there were 423 patients (44.5%) in the elderly group. Worse neurological function was observed in the elderly group than in the younger group; the elderly group had higher NIHSS and mRS scores and lower BI. However, there were no significant differences in stroke severity and subtype between the age groups (all *P* > 0.05). Moreover, the levels of TC, TG, HDL-C, LDL-C, FG, and HbA1c were not significantly different between the two groups (Table 1).

**Table 1:** Clinical features and previous history of diseases in patients with ischemic stroke and AF by age group

Characteristics	Young group	Elderly group	*P*
Cases, n (%)	528 (55.5)	423 (44.5)	—
Gender, n (%):			0.271
Men	296 (57.1)	222 (42.9)	
Women	232 (53.6)	201 (46.4)	
OCSP classification, n (%):			0.195
Partial anterior circulation infarct	327 (62.5)	236 (56.3)	
Total anterior circulation infarct	91 (17.4)	88 (21.0)	
Lacunar infarct	9 (1.7)	12 (2.9)	
Posterior circulation infarct	96 (18.4)	83 (19.8)	
Stroke severity, n (%):			0.691
Mild	211 (40.0)	162 (38.3)	
Moderate	145 (27.5)	112 (26.5)	
Severe	172 (32.6)	149 (35.2)	
Neurological function:			
NIHSS	10 (15)	12 (14)	0.005
BI	30 (60)	20 (50)	0.001
mRS	4 (3)	4 (2)	0.002
Laboratory examination:			
Total cholesterol:	4.91 (1.18)	4.84 (1.24)	0.434
Triglyceride:	1.35 (0.89)	1.26 (1.03)	0.162
High density lipoprotein cholesterol:	1.15 (0.40)	1.16 (0.29)	0.526
Low density lipoprotein cholesterol:	3.08 (0.96)	2.97 (0.96)	0.093
Fasting glucose	6.55 (2.68)	6.64 (2.86)	0.677
Glycosylated hemoglobin	6.34 (0.96)	6.29 (1.27)	0.671

### Differences in stroke risk factors and lifestyle factors between groups

There were no obvious differences in the prevalence of hypertension, DM, dyslipidemia, obesity, and current smoking, but there was a higher frequency of alcohol consumption in the younger group than in the elderly group (10.4% vs. 5.0%, *P* = 0.002) (Table 2).

**Table 2:** Clinical features and previous history of diseases in patients with ischemic stroke and AF by age group

Risk Factors	Young group	Elderly group	*P*
Hypertension	340 (64.4)	276 (65.2)	0.784
Diabetes	144 (27.3)	94 (22.2)	0.074
Dyslipidemias	136 (25.8)	100 (23.6)	0.452
Artery stenosis	85 (16.1)	87 (20.6)	0.075
Obesity	73 (13.8)	67 (15.8)	0.384
Current smoking	129 (24.4)	98 (23.2)	0.650
Alcohol consumption	55 (10.4)	21 (5.0)	0.002

### Differences in outcomes at 1 and 3 years after stroke between groups

Table 3 shows that poor outcomes were observed in the elderly group. The mortality rate was higher in the elderly group than in the young group (OR [95% CI], 1.86 [1.36-2.54] 1 year after stroke, and 2.54 [1.83-3.53] at 3 years after stroke, all *P* < 0.001). Similar trends were observed for dependency rates (OR [95% CI], 1.85 [1.40-2.44] at 1 year after stroke, and 2.80 [1.81-4.35] at 3 years after stroke, all P < 0.001). Recurrence rates were significantly higher in the elderly group than in the young group at 3 years after stroke (OR [95% CI], 1.76 [1.14-2.ck=“SelectText(‘bib72’);return true;”>72], *P* < 0.001). However, the significant difference in recurrence rates disappeared after adjusting for stroke subtype, severity, risk factors, and lifestyle factors. The OR (95% CI) for mortality rate was 1.98 (1.39–2.82, *P* < 0.001) at 1 year and 2.95 (2.02–4.32, *P* < 0.001) at 3 years; the corresponding values for dependency rates were 1.79 (1.33–2.42, *P* < 0.001) and 3.06 (1.90–4.93, *P* < 0.001), respectively.

**Table 3:** Outcomes and odds ratios 1 year and 3 years after stroke among patients with AF by age group

Outcomes	Young group	Elderly group	Univariate analysis		Multivariate analysis	
			OR(95%CI)	*P*	OR(95%CI)	*P*
1 year:						
Mortality	98 (20.5)	118 (32.4)	1.86 (1.36, 2.54)	<0.001	1.98 (1.39, 2.82)	<0.001
Dependency	224 (47.0)	226 (62.1)	1.85 (1.40, 2.44)	<0.001	1.79 (1.33, 2.42)	<0.001
Recurrence	81 (21.3)	62 (25.6)	1.27 (0.87, 1.86)	0.214	1.16 (0.78, 1.71)	0.475
3 years:						
Mortality	104 (29.7)	145 (51.8)	2.54 (1.83, 3.53)	<0.001	2.95 (2.02, 4.32)	<0.001
Dependency	257 (73.4)	248 (88.6)	1.76 (1.14, 2.72)	<0.001	1.57 (0.98, 2.51)	<0.001
Recurrence	122 (52.1)	90 (65.7)	2.80 (1.81, 4.35)	<0.001	3.06 (1.90, 4.93)	0.011

## DISCUSSION

This is the first report on the effects of age on long-term outcomes among stroke patients with AF in China. We compared differences in clinical features, conventional stroke risk factors, and outcomes at 1 year and 3 years after stroke between younger and elderly patients.

Previous studies indicated that stroke patients with AF were less likely than stroke patients without AF to have conventional risk factors for stroke, including hypertension, current smoking and alcohol consumption habits, and diabetes [21, 22]. Other studies reported significant differences in the frequency of conventional stroke risk factors [23]. The present study focused on patients with stroke and AF and showed that the prevalence of hypertension, diabetes, dyslipidemias, arterial stenosis, obesity, and current smoking were not significantly different between the two age groups. However, the frequency of alcohol consumption was greater in the younger group than in the elderly group. At the same time, we did not observe any differences in the levels of TC, TG, HDL-C, LDL-C, FG, and HbA1c between the younger and elderly groups. These results indicate that there is not an obvious relationship between age and the prevalence of conventional risk factors in stroke patients with AF.

Moreover, there remains controversy regarding differences in outcomes after stroke between patients with and without AF in the elderly, especially with respect to the long-term outcomes for elderly stroke patients with AF. Many studies have demonstrated that elderly stroke patients were more likely to have poor outcomes after acute ischemic stroke, including increased short-term and long-term mortality rates, dependency rates, and risk of stroke recurrence [15–19, 24, 25]. Previous studies have similarly reported that AF is associated with higher mortality rates [26–29], higher recurrence rate [26, 30], and a markedly increased dependency in stroke patients with AF [31]. However, long-term outcomes for elderly stroke patients with AF remain uncertain, and there is in particular a relative paucity of longitudinal data that assess long-term outcomes after stroke in China. In the present study, we found that mortality and dependency rates were significantly higher in the elderly group than in the younger group at both 1 and 3 years after stroke in patients with AF. There was a higher observed recurrence rate at 3 years after stroke in the elderly group than in the younger group. One-third of patients in the younger group and more than one-half of patients in the elderly group had died by 3 years after stroke onset. Moreover, most survivors experienced dependency (73.4% in the younger group and 88.6% in the elderly group) and recurrence (52.1% in the younger group and 65.7% in the elderly group) 3 years after stroke onset. These trends did not change after adjustment using clinical features, risk factors, and lifestyle factors as covariates. There was an incremental increase in the risk of mortality and dependency, but not recurrence. Suboptimal treatment for AF in Chinese patients compared with that for Western patients with AF may explain the poor outcomes after stroke in elderly patients [32–34].

Moreover, there were several limitations in this study. First, this study used a local stroke registry, and all patients were recruited from same city; thus, limited representation occurred. Second, patients who died before admission to the stroke unit and those lost to follow-up were excluded in this study, so poor outcomes may be underestimated. Third, pre-stroke data were lacking, including treatment and medicine use for AF.

## CONCLUSIONS

In this large hospital-based stroke registry, we compared differences in clinical features, risk factors, and outcomes at 1 and 3 years following AF-associated stroke between younger and elderly patients. There was a higher prevalence of AF in the elderly group than in the younger group. The prevalence of hypertension, DM, dyslipidemia, artery stenosis, obesity, and current smoking were not obviously different between the two groups, but there was a higher frequency of alcohol drinking in the younger group. Poorer long-term outcomes were observed in the elderly group. There was an incremental risk of mortality and dependency at 1 and 3 years after stroke in the elderly group. These findings suggest that elderly stroke patients, especially those with AF, should be targeted for secondary stroke prevention to reduce the burden due to stroke in China.

## MATERIALS AND METHODS

### Patient selection

This was a hospital-based follow-up study using the Stroke Registry System. All consecutive patients with acute ischemic stroke admitted to the stroke unit within 72 hours of stroke onset in three hospitals in Tianjin, China, between January 2006 and September 2014 were recruited to this study. All patients were diagnosed according to World Health Organization criteria confirmed via brain computed tomography (CT) or magnetic resonance imaging (MRI) [35]. Patients experiencing transient ischemic attack were excluded from this study. Trained neurologists selected detailed information for patients with acute ischemic stroke, including ischemic stroke subtype, stroke severity, previous medical history, lifestyle factors, and outcomes at 1 year and 3 years after stroke.

To ensure data quality, three groups of senior trained neurologists (the assessment group, the follow-up group, and the quality control group) were responsible for determining the nervous system score at admission, for the reexamination (including of neurological score, risk factor management, and directing the treatment and rehabilitation) during follow-up, and a sampled confirmation of 20% of all patients each month, respectively.

All investigative protocols were approved by the ethics committee of Tianjin Medical University General Hospital. The methods were carried out in accordance with approved guidelines, and informed consent was obtained from each patient.

### Stroke subtypes

Stroke subtypes, which were classified on admission, included total anterior circulation infarct (TACI), partial anterior circulation infarct (PACI), lacunar infarct (LACI), and posterior circulation infarct (POCI), according to the Oxfordshire Community Stroke Project (OCSP) criteria [36].

### Stroke severity

Stroke severity was categorized into three categories according to the National Institutes of Health stroke scale (NIHSS): mild (NIHSS score ≤7), moderate (NIHSS score 8–16), and severe (NIHSS score ≥17) [37]. Meanwhile, the Bethel index (BI) and mRS scores were also recorded on admission.

### Stroke risk factors and lifestyle factors

Risk factors for stroke included hypertension, diabetes mellitus, dyslipidemia, AF, brain-supplied arterial stenosis, and obesity (body mass index [BMI] ≥30 kg/m^2^). Lifestyle factors included current smoking (≥1 cigarette per day for ≥1 year) and alcohol consumption (drinking alcohol at least 1 time per week for 1 year).

The levels of total cholesterol (TC), triglycerides (TG), high-density lipoprotein cholesterol (HDL-C), low-density lipoprotein cholesterol (LDL-C), fasting glucose (FG), and glycosylated hemoglobin (HbA1c) were recorded on admission.

### Outcomes after stroke

Outcomes after stroke were described by mortality, recurrence, and dependency at 1 and 3 years after stroke. Outcomes were assessed with both face-to-face and telephone follow-up. Death was defined as all-cause cumulative death in corresponding periods after stroke and was determined based on reports from patients’ family members and telephone follow-up. Stroke recurrence was defined as all new-onset vascular events, including stroke, myocardial infarction, and venous thrombosis, 30 days after stroke. Information pertaining to diagnosis and classification of recurrent stroke were obtained from the medical record department in the discharge hospitals. Dependency was defined as an mRS score >2 [20].

### Statistical analysis

All patients with acute ischemic stroke were classified into two groups based on age (<75 years and ≥75 years) for analysis. Continuous variables, including age, NIHSS score, BI, mRS score, and TG, TC, HDL-C, LDL-C, FG, and HbA1c levels were presented as mean (standard deviation) or median (range), as appropriate, and were compared between the two groups using the Student t-test or the Mann-Whitney U-test. Dichotomous variables, including stroke subtype, stroke severity, stroke risk factors, and outcomes over different periods after stroke, were presented as numbers (percentages) and were compared with the chi-squared test. Differences in outcomes between the two groups were assessed with a logistic regression analysis. A multivariate analysis was carried out with a logistic regression model to evaluate differences in outcomes over different periods after stroke, with stroke subtype, stroke severity, risk factors, and lifestyle factors as covariates. Data for this analysis were presented as adjusted odds ratios (ORs) with 95% confidence intervals (CIs). Patients lost to follow-up were excluded from outcome evaluation. All statistical analyses were performed using SPSS version 19.0 (SPSS Inc., Chicago, IL), and a two-tailed *P* < 0.05 indicated statistical significance.
